# High Level of Anaphylatoxin C5a Predicts Poor Clinical Outcome in Patients with Clear Cell Renal Cell Carcinoma

**DOI:** 10.1038/srep29177

**Published:** 2016-07-06

**Authors:** Wei Xi, Li Liu, Jiajun Wang, Yu Xia, Qi Bai, Qilai Long, Yiwei Wang, Jiejie Xu, Jianming Guo

**Affiliations:** 1 Department of Urology, Zhongshan Hospital, Fudan University, Shanghai 200032, China; 2 Department of Biochemistry and Molecular Biology, School of Basic Medical Sciences, Fudan University, Shanghai 200032, China

## Abstract

Anaphylatoxin C5a, a potent pro-inflammatory peptide produced in the process of complement activation, was proved to have a vital role in tumor initiation and progession by previous investigations. However whether it could act as a prognostic marker remains unknown. Here we retrospectively enrolled 272 ccRCC patients undergoing nephrectomy in Zhongshan Hospital, Shanghai between 2005 and 2007. C5a level was assessed by immunohistochemistry and its association with clinicopathologic features and prognosis were evaluated. Our results indicated that high tumoral C5a level was associated with poor overall survival (OS) (hazard ratio = 1.753, 95% CI 1.068–2.878, P = 0.026). In addition, tumoral C5a could significantly stratify patients’ prognosis both in advanced stage (TNM III + IV) and intermediate/high risk group (SSIGN score ≥4) (*P* < 0.001 and = 0.008, respectively). Furthermore, incorporating tumoral C5a with other parameters could improve the predicting accuracy, compared with TNM and SSIGN system (c-index = 0.789, 0.713 and 0.727, respectively). In conclusion, tumoral C5a is an independent adverse prognostic biomarker for clinical outcome of ccRCC patients after nephectomy.

Renal cell carcinoma (RCC), arising from renal tubular epithelial cells, accounts for approximate 2–3% of all malignancies in adults^1^. In China, over 54 out of 100,000 people are diagnosed with RCC every year^2^, and most of the cases (70–85%) are clear cell RCC (ccRCC), histologically^3^. Although incidental RCC has taken up more than 50% of all RCCs due to the widespread use of abdominal imaging^4^,^5^, 25–30% of the patients still have the initial diagnosis with metastasis^6^. Moreover, another 20% of the patients will have a relapse and develop metastatic RCC (mRCC) after curative nephrectomy^7^. Characterized by the resistance to radiotherapy and chemotherapy, mRCC exhibits an extremely poor prognosis^8^,^9^, which makes the predicting system more important.

Currently, some clinical or pathological predicting systems are used to predict outcomes of RCC patients. TNM stage and Fuhrman grade are traditional, but the most common used systems. Meanwhile, integrated systems, including Mayo Clinic stage, size, grade and necrosis (SSIGN) score and University of California integrated Staging System (UISS), are also clinically employed^10^,^11^. With these predicting systems, however, accurate prediction remains difficult, thus better or novel predictors of survival of RCC are needed^10^,^12^.

Inflammatory microenvironment plays a vital role during tumor initiation and progression^13^. Renal cell carcinoma also exhibits a strong connection with hypoxia and enhanced inflammatory signaling^14^. Anaphylatoxin C5a, including C5a itself and its spliced form C5adesArg- being removed of the C-terminal arginine, is another potent pro-inflammatory peptide produced in the process of complement activation^15^. Growing evidence has suggested that C5a may serve as a negative wrecker in tumor initiation and progression by modulating microenvironment^16^,^17^. Additionally, it is reported that high level of C5a receptor (C5aR) is associated with poor clinical outcome in lung cancer^18^, while no investigations have focused on the prognostic function of C5a. Therefore, we are curious about the association between C5a and ccRCC patients’ prognosis and speculate that C5a could probably function as a prognostic marker.

In this study, we sought to identify the clinical and prognostic value of C5a in ccRCC by immunohistochemical staining. We analysed the association of tumoral C5a level with clinicopathological characteristics and clinical outcomes (OS and RFS). Moreover, C-index was applied to explore the effect of tumoral C5a on prediction accuracy by incorporating it with traditional systems.

## Results

### Patient characteristics and immunohistochemical findings

The major characteristics of this cohort were shown in Table 1. The median follow-up was 99.0 months (2.6–120.5months) and the median age was 55 years old (15–83 years). Generally, most of the patients were male (69.1%), and tumors were predominantly of well differentiation (Fuhrman grade 1 + 2, 84.2%) and early stage (TNM I + II, 69.9%), and good physical status(ECOG-PS score = 0) prevailed (73.2%). 16 out of 272 (5.9%) patients were identified with lymph node or distant metastasis.

**Figure 1:**
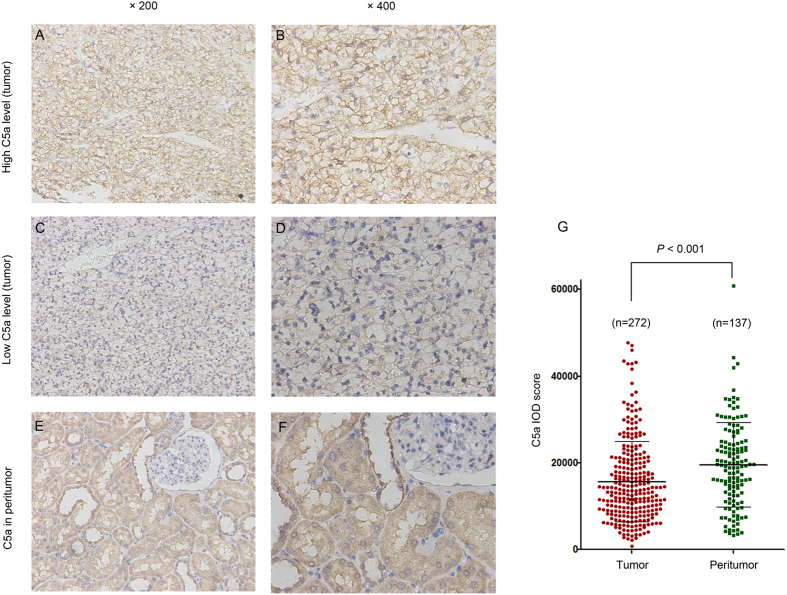
Immunohistochemical analyses of C5a in ccRCC specimens. Representative high C5a level in tumor tissues with (**A**) ×200 magnification, and (**B**) ×400 magnification; representative low C5a level in tumor tissues with (**C**) ×200 magnification, and (**D**) ×400 magnification; representative C5a staining in peritumor tissues with (**E**) ×200 magnification, and (**F**) ×400 magnification; (**G**) IOD score of C5a level in tumor and non tumor tissue. The three horizontal lines represent mean C5a and ±standard error of mean (SEM). P-value, calculated by Mann Whitney test, <0.05 was regarded as statistically significant.

To assess the intensity of C5a in ccRCC patients, anti-C5a IHC staining was performed on tumoral and peritumoral tissues. According to the staining intensity and corresponding IOD score, we divided the tumoral C5a level into high and low groups by X tile (Fig. 1A–D). C5a staining predominantly located on the membrane of tumor cells and extracellular matrix in tumor tissues. In peritumoral tissues, C5a was diffusely distributed in the entire tubular epithelial cells with glomerulus merely being stained (Fig. 1E,F). Albeit the higher IOD score and wider range (mean ± SD: 19530 ± 9741 in peritumor vs 15699 ± 9255in tumor, respectively, *P* < 0.001, Fig. 1G), we ruled out the necessity of further analyzing peritumoral C5a in its prognostic properties, as the minimum *P* value was 0.75 by X tile.

### Associations between C5a and clinicopathological parameters

We further evaluated the associations between clinicopathological features and tumoral C5a level. As shown in Table 1, C5a was only significantly correlated with necrosis in tumors of this cohort (*P* = 0.026). There was no significant relationship with any other clinicopathological characteristics.

### Prognostic significance of C5a

**Figure 2:**
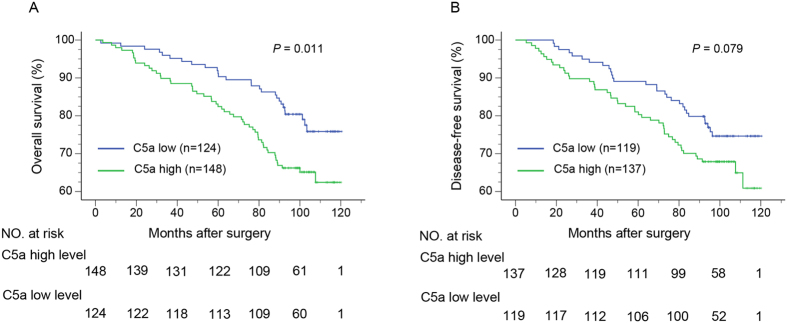
Kaplan-Meier analyses for OS and DFS of ccRCC patients according to tumoral C5a level. (**A**) OS according to tumoral C5a level and; (**B**) DFS according to tumoral C5a level.

In order to assess whether tumoral C5a has potential significance in predicting clinical outcome of ccRCC patients, Kaplan-Meier analyses were applied to compare OS and DFS in different C5a leveled patients. Compared with those in low level, patients with high level of C5a exhibited a significant poorer OS (*P* = 0.011; Fig. 2A). However, the two groups divided by C5a were of no statistical difference in DFS-probably due to the limited specimens, since the separating trend could be observed (*P* = 0.079; Fig. 2B). Therefore, DFS was excluded in the next stratifying analyses.

**Figure 3:**
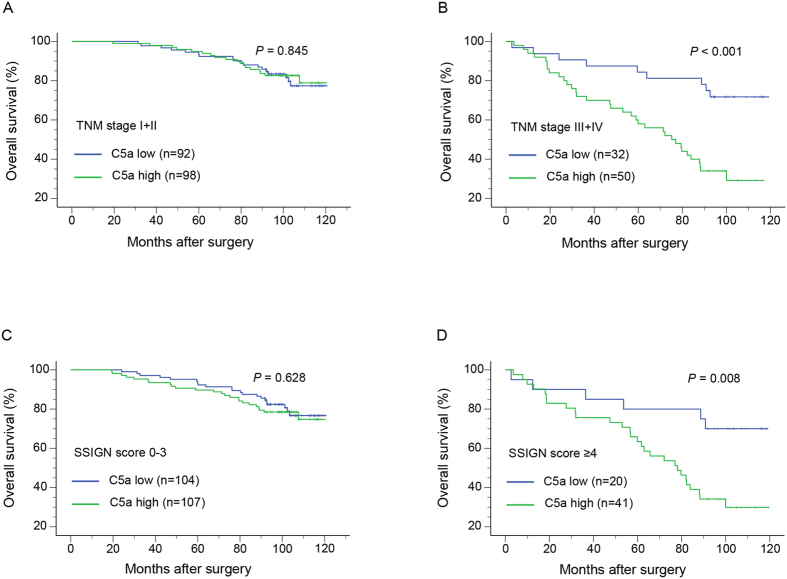
Kaplan-Meier analyses for OS in ccRCC according to tumoral C5a level stratified by TNM stage and SSIGN score. Kaplan-Meier analyses for OS of ccRCC patients according to C5a level in (**A**) early-stage (TNM I + II), (**B**) advanced-stage (TNM III + IV), (**C**) low risk (SSIGN score 0–3), (**D**) intermediate/high risk (SSIGN score van stratified groups.

To ascertain what exquisite subgroups the potential prognostic value lied in, we chose TNM stage and SSIGN system to stratify the patients, because TNM stage is the most common used parameter and SSIGN a classical comprehensive system. As the results showed in Fig. 3A,B, high level of C5a strongly correlated with shorter OS in advanced stage (TNM III + IV) subgroups (*P* < 0.001, Fig. 3B). While discrepancy was not observed in early stage patients (*P* = 0.845, Fig. 3A). Patients were used to be stratified into low/intermediate/high risk groups as the clinical outcomes were completely different^11^. However, considering the limited high risk patients (SSIGN score he l6/272, 2.2%) in this cohort, we combined intermediate (SSIGN score 4–7) and high risk patients into one intermediate/high risk group (SSIGN score ≥4). As shown in Fig. 3C,D, patients with high C5a level was also an adverse prognostic factor for OS in intermediate/high risk subgroup (*P* = 0.008; Fig. 3D), while curves in low risk (SSIGN score 0–3) subgroup were of no difference (*P* = 0.628; Fig. 3C).

Further, univariate and multivariate analyses were conducted to identify the independence of C5a. Parameters with statistical significance in univariate analyses were brought into the multivariate analyses. But we excluded single T, N and M classification in the multivariate analyses in case of overlap with TNM stage variable. As shown in Table 2, univariate analysis reconfirmed the strong significance between C5a and OS (HR 1.818, 95% CI 1.142–2.894, *P* = 0.012), and multivariate analysis verified the independence of C5a in predicting ccRCC patients’ OS (HR 1.753, 95% CI 1.068–2.878, *P* = 0.026), just like TNM stage (*P* < 0.001), Fuhrman grade (*P* = 0.002), Necrosis (*P* = 0.023), and ECOG-PS (*P* < 0.001).

Collectively, these data demonstrated that C5a level was an independent prognostic factor in ccRCC patients for predicting OS, and it performed better in advanced stage and intermediate/high risk patients.

### Construction of a novel integrated system and accuracy evaluation

**Figure 4:**
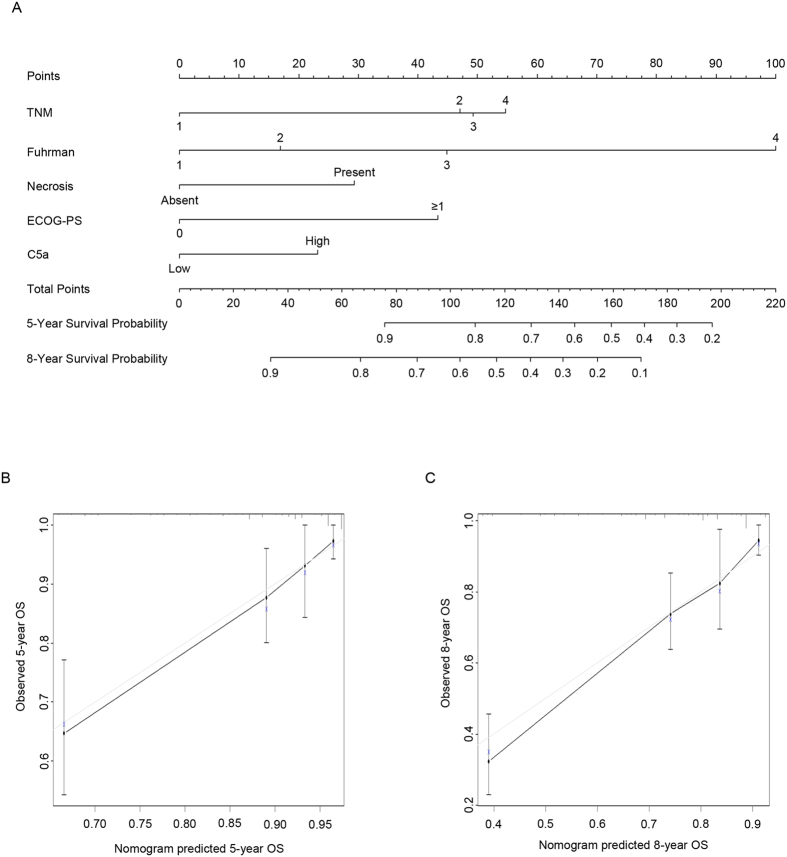
Nomogram for predicting 5- and 8-year OS in ccRCC patients. (**A**) Nomogram for predicting clinical outcomes integrating tumoral C5a level, TNM stage, Fuhrman grade and necrosis; (**B**) calibration plot for predicted and observed 5-year overall survival rate; and (**C**) calibration plot for predicted and observed 8-year overall survival rate. The gray line: ideal model, vertical bars: 95% CI, and ECOG-PS = Eastern Cooperative Oncology Group performance status.

We then sought to integrate the independent characteristics in the multivariate analysis into one system. To achieve this goal, we set up a nomogram for OS, and C5a was obviously a negative factor in the nomogram (Fig. 4A). Boottrap validation was performed for calibration of 5- and 8-year survival (Fig. 4B,C). To precisely evaluate the accuracy, we compared the c-index of the novel system with that of TNM stage and SSIGN system. The c-index of our integrated system, TNM stage and SSIGN system was 0.789, 0.713 and 0.727, respectively. These data suggested that the novel integrated system was more accurate than traditional TNM grade and SSIGN system in predicting OS of ccRCC patients.

## Discussion

Early staged RCC is a curable disease as the 5-year overall survival rate of patients in early stage (TNM I + II) is about 91% after surgery^7^,^19^. However, circumstance changes when it comes to advanced stage (TNM III + IV) patients, as the figure precipitates to 59% in stage III and 20% in stage IV^7^. Therefore, sharpening the prognostic systems in predicting the clinical outcome, especially outcome of advanced stage patients, seems much more worthwhile. In this study, we for the first time, proposed a new independent biomarker C5a, which was significantly associated with overall survival of ccRCC patients, especially in TNM advanced stage and SSIGN intermediate/high risk patients. Integrating it with other parameters exhibited a more accurate prediction.

Inflammation plays a crucial role in cancer and could provide potential therapeutic targets. During initiation and progression of tumors, produced inflammatory factors (cytokines, chemokines, etc) could provoke the activating, infiltrating, and trafficking of various inflammatory immune cells into tumor tissue^14^,^20^ to form a tumor-promoting environment. In kidney cancer, several associated inflammatory signaling pathways (VHL, mTOR, TNF, STAT) was proved to be dramatically associated with carcinogenesis^14^, and targeting vital molecules in these pathways attenuates tumor growth and progression, based on which new drugs are developed^14^,^21^,^22^,^23^. C5a, a 74-amino-acid glycopeptide produced by the enzymatic cleavage of C5, is a potent inflammatory mediator and chemoattractant that has been reported to be able to regulate anti-tumor response^16^,^17^,^24^. The fact that tumor cells themselves could not only enhance the production of C5a, but also express protecting protein to avoid complement induced lysis suggests a membrane attacking complex (MAC)-independent way^16^,^25^. That hypothesis is directly or indirectly supported by many investigations^16^,^17^,^26^,^27^. This indirect antitumor effect could be abrogated by C5aR knock down or treatment of C5aR antagonist, indicating an important role of C5a/C5aR in tumor microenvironment^16^,^17^. Therefore C5a/C5aR axis is a potential therapeutic target in cancer^28^.

Limitations still exist in our study. First of all, it is a single retrospective cohort with limited patients. We are excited and confident in our principal findings- high C5a level predicts poor prognosis- as it is consistent with the argument of several previous rigorous basic scientific investigations^16^,^17^,^29^. It was the significant correlations between C5a and OS that we conclude from. However, C5a was not associated with DFS statistically in our study, and we are inclined to attribute this insignificance to the limited patients of our cohort since a separating tendency could be observed and statistical significance should appeared if we only performed Kaplan-Meier analyses in advanced stage patients (Figure not shown). Therefore we speculate that C5a could be more efficient in pure high risk patients such as advanced stage, even metastatic RCC (mRCC) patients. Actually we have been working on mRCC patients’ follow-up, and our next clinical investigation are to focus on the role of tumoral C5a in mRCC patients. Secondly, our study was based on the IHC staining and score, which would be more persuasive by adjuvant measurement of relative mRNA expression, such as complement regulatory gene expression. However, although we are collecting fresh tissue samples for mRNA evaluation, not enough is prepared for the time being. Last but not least, complement is a complicated system involving dozens of proteins, one complement C5a does not represent the entire family. More investigations, especially mechanism researches, are to help with deeper understanding and potential clinical utilization of complement system.

All in all, our study demonstrate that C5a was an adverse prognostic factor in ccRCC patients, and further more investigations are supposed to carry on.

## Conclusions

Tumoral C5a is a novel prognostic marker in ccRCC patients. High tumoral C5a level is associated with OS, and it is better performed in TNM advanced stage and SSIGN intermediate/high risk patients. Integrating tumoral C5a with other clinical parameters could increase the predicting accuracy, which provides an alternative postoperational surveillance system.

## Materials and Methods

### Patients

A total of 272 patients with pathologically validated ccRCC after partial or radical nephrectomy, between Feb 2005 and Jun 2007, were enrolled from the Department of Urology, Zhongshan Hospital, Fudan University. All methods mentioned in this article were approved by the ethics committee of Zhongshan Hospital (approval number B2015-030) and were carried out in accordance with the approved guidelines and regulations (REMARK criteria). Written informed consent on the use of clinical specimens from each patient was achieved. The inclusion criteria were: no history of other malignant tumors, no history of anticancer therapy, pathologically proven ccRCC, and patients after radical or partial nephrectomy. The exclusion criteria were: mixed histological type of primary renal cancer, tumor necrosis area over 80%, and patients died within the first month after surgery. Patients were followed up every 3 months, and finally ended on January 30, 2015. Patients’ basic information (age, gender, ECOG-PS), tumor pathological information (tumor size, Fuhrman grade, necrosis, TNM stage), and survival or recurrence information (date of death or recurrence, or last follow-up) were recorded. Tumor size was faithfully recorded as the longest diameter described in pathology report. Necrosis, histological type and Fuhrman grade were reassessed by two independent pathologists. Presence of nodal and metastasis was defined according to intraoperative, pathologic, and radiographic findings. With radiographic reports and postoperative data, we reassigned the stage according to the 2010 AJCC TNM classification^30^, and final TNM stage was confirmed by one urologist. OS was defined as the time span from curative surgery to death of any cause. DFS was defined as the time span from curative surgery to recurrence or metastasis. None of the patients in this cohort has ever received targeted therapies before or after surgery.

### Immunohistochemistry and evaluation

Tissue microarrays (TMA) were established as previously described^31^. Primary monoclonal anti-C5a antibody (1:100 dilution, ab11878, Abcam, Cambridge, MA, USA) were applied in the procedure. Three independent shots of C5a staining areas (away from tissue margin, obvious inflammatory and necrotic domains) were taken for analysis. The intensity was analyzed by Image-Pro Plus 6.0 and integrated optical intensity (IOD), representing the staining intensity, was recorded. The TMA slides were observed and evaluated by two investigators unaware of the clinical information. To determine the optimum cutoff IOD score, we performed X tile plot analysis with X tile software 3.6.1, and select the cut point by the rule of “minimum *P* value”. The cutoff point was 12578 for tumoral C5a staining, but not a significant point was found in peritumoral C5a staining.

### Statistical analyses

GraphPad Prism 6, SPSS 19.0 and Stata 12.0 were used to do the statistical analyses of this study. *P*-value < 0.05 was regarded as statistically significant. Mann-Whitney test was applied to compare the tumoral and peritumoral IOD score. Fisher’s exact method, χ^2^ test, or Cochran-Mantel-Haenszel χ^2^ test were applied to analyze the associations between staining level and patients’ clinical features. Kaplan-Meier analyses and log-rank tests were applied for OS and DFS evaluation. Univariate and multivariate Cox proportional hazard models were applied to evaluate the HR and 95% CI. Further nomogram construction and calibration were performed with R software 3.0.2 with the “rms” package. Finally, we compared the Harrell’s concordance index (c-index) of different predicting systems to evaluate the prognostic accuracy.

## Additional Information

**How to cite this article**: Xi, W. *et al*. High Level of Anaphylatoxin C5a Predicts Poor Clinical Outcome in Patients with Clear Cell Renal Cell Carcinoma. *Sci. Rep.* **6**, 29177; doi: 10.1038/srep29177 (2016).

## Figures and Tables

**Table 1:** Association between C5a and clinicopathologic characteristics.

Characteristics	Patients		C5a level		
	n	%	low	high	*P*-value
All patients	272	100	124	148	
Age, years*	range	15–83			0.150^†^
≤55	134	49.3	67	67	
>55	138	50.7	57	81	
Gender					0.133^†^
Female	84	30.9	44	40	
Male	188	69.1	80	108	
Tumor size, cm*					0.155^†^
≤4	154	56.6	76	78	
>4	118	43.4	48	70	
T classification					0.063^‡^
T1	168	61.8	83	85	
T2	22	8.1	9	13	
T3	64	23.5	27	37	
T4	18	6.6	5	13	
N classification					0.527^‡^
N0	261	95.9	120	141	
N1	11	4.1	4	7	
Distant metastasis					0.446^†^
No	258	94.9	119	139	
Yes	14	5.1	5	9	
TNM stage					0.274^‡^
I	168	61.8	83	85	
II	22	8.1	9	13	
III	64	23.5	27	37	
IV	18	6.6	5	13	
Fuhrman grade					0.149^‡^
1	29	10.7	15	14	
2	200	73.5	95	105	
3	40	14.7	12	28	
4	3	1.1	2	1	
Necrosis					**0.026**^†^
Absent	234	86.0	113	121	
Present	38	14.0	11	27	
ECOG-PS					0.195^†^
0	199	73.2	86	113	
≥1	73	26.8	38	35	

*Split at median; ^†^χ^2^ test or Fisher’s exact test, ^‡^Cochran-Mantel-Haenszel χ^2^ test, P-value < 0.05 was regarded as statistically significant; ECOG-PS = Eastern Cooperative Oncology Group performance status.

**Table 2:** Univariate and multivariate analyses of characteristics associated with overall survival.

Characteristics	Univariate			Multivariate		
	Hazard Ratio	95%CI	*P-value*^†^	Hazard Ratio	95%CI	*P-value*^†^
Age, years*						
>55 *vs* ≤55	2.012	1.269–3.192	0.003	1.497	0.928–2.412	0.098
Gender						
Male *vs* Female	1.039	0.643–1.679	0.874	–	–	–
T classification			<0.001			–
T1	1.000	reference	–	–	–	–
T2	3.703	1.929–7.109	<0.001	–	–	–
T3	3.606	2.196–5.921	<0.001	–	–	–
T4	8.053	2.443–26.550	0.001	–	–	–
N classification						
N1 *vs* N0	1.058	0.139–8.063	0.001	–	–	–
Distant metastasis						
Yes *vs* No	5.739	3.030–10.870	<0.001	–	–	–
TNM stage			<0.001			**<0.001**
I	1.000	reference		1.000	reference	–
II	3.557	1.713–7.383	0.001	3.190	1.501–6.778	0.003
III	3.821	2.248–6.496	<0.001	3.611	2.058–6.334	<0.001
IV	10.340	5.383–19.861	<0.001	5.856	2.867–11.964	<0.001
Tumor size						
>4 cm *vs* ≤4 cm	2.137	1.363–3.350	0.001	1.032	0.600–1.775	0.910
Fuhrman grade			<0.001			0.002
1–2	1.000	reference	–	1.000	reference	–
3	2.864	1.741–4.711	<0.001	1.743	1.023–2.969	0.041
4	4.177	1.305–13.368	0.016	7.060	2.054–24.264	0.002
Necrosis						
Present *vs* Absent	2.760	1.673–4.553	<0.001	1.902	1.092–3.312	0.023
ECOG-PS						
≥1 *vs* 0	3.236	2.080–5.036	<0.001	2.422	1.500–3.913	<0.001
C5a level						
High *vs* Low	1.818	1.142–2.894	0.012	1.753	1.068–2.878	0.026

*Split at median; ECOG-PS = Eastern Cooperative Oncology Group performance status; CI = confidence interval; OS = overall survival; DFS = disease free survival; ^†^Data obtained from the Cox proportional hazards model, P-value < 0.05 was regarded as statistically significant.
